# Duration of effectiveness of the COVID-19 vaccine in Japan: a retrospective cohort study using large-scale population-based registry data

**DOI:** 10.1186/s12879-024-09488-6

**Published:** 2024-06-28

**Authors:** Kohei Uemura, Sachiko Ono, Nobuaki Michihata, Hayato Yamana, Hideo Yasunaga

**Affiliations:** 1 Department of Biostatistics & Bioinformatics, Interfaculty Initiative in Information Studies, The University of Tokyo, 7-3-1 Hongo, Bunkyo-ku, Tokyo, 113-8655 Japan; 2 Department of Eat-loss Medicine, Graduate School of Medicine, The University of Tokyo, Tokyo, Japan; 3 Cancer Prevention Center, Chiba Cancer Center Research Institute, Chiba, Japan; 4 Data Science Center, Jichi Medical University, Shimotsuke, Japan; 5 Department of Clinical Epidemiology & Health Economics, School of Public Health, Graduate School of Medicine, The University of Tokyo, Tokyo, Japan

**Keywords:** Administrative claims data, Cohort study, COVID-19 vaccination, Time-dependent effectiveness

## Abstract

**Background:**

Most evidence of the waning of vaccine effectiveness is limited to a relatively short period after vaccination.

**Methods:**

Data obtained from a linked database of healthcare administrative claims and vaccination records maintained by the municipality of a city in the Kanto region of Japan were used in this study. The study period extended from April 1, 2020, to December 31, 2022. The duration of the effectiveness of the COVID-19 vaccine was analyzed using a time-dependent piecewise Cox proportional hazard model using the age, sex and history of cancer, diabetes, chronic obstructive pulmonary disease, asthma, chronic kidney disease, and cardiovascular disease as covariates.

**Results:**

Among the 174,757 eligible individuals, 14,416 (8.3%) were diagnosed with COVID-19 and 936 (0.54%) were hospitalized for COVID-19. Multivariate analysis based on the time-dependent Cox regression model with reference of non-vaccine group revealed a lower incidence of COVID-19 in the one-dose group (hazard ratio, 0.76 [95% confidence interval, 0.63–0.91]), two-dose (0.89 [0.85–0.93]), three-dose (0.80 [0.76–0.85]), four-dose (0.93 [0.88–1.00]), and five-dose (0.72 [0.62–0.84]) groups. A lower incidence of COVID-19-related hospitalization was observed in the one-dose group (0.42 [0.21–0.81]), two-dose (0.44 [0.35–0.56]), three-dose (0.38 [0.30–0.47]), four-dose (0.20 [0.14–0.28]), and five-dose (0.11 [0.014–0.86]) groups. Multivariable analyses based on the time-dependent piecewise Cox proportional hazard model with reference of non-vaccine group revealed significant preventive effects of the vaccine for 4 months for the incidence of COVID-19 and ≥ 6 months for hospitalization.

**Conclusions:**

Vaccine effectiveness showed gradual attenuation with time after vaccination; however, protective effects against the incidence of COVID-19 and hospitalization were maintained for 4 months and ≥ 6 months, respectively. These results may aid in formulating routine vaccination plans after the COVID-19 pandemic.

**Supplementary Information:**

The online version contains supplementary material available at 10.1186/s12879-024-09488-6.

## Background

High vaccination coverage against coronavirus disease 2019 (COVID-19) has contributed to the end of the pandemic. However, continuous measures must be taken against the outbreak of epidemics, including the emergence of new mutant strains. The “Omicron JN.1” strain, a descendant of the Omicron BA.2.86 strain, has spread globally. The duration and frequency of the of booster doses need to be established. A better understanding of the waning of vaccine-induced protection plays an important role in formulating post-pandemic vaccination plans.

Several COVID-19 types of vaccines have been manufactured worldwide since December 2020; however, clinical and laboratory evidence accumulated over a short period has shown that the effectiveness of the COVID-19 vaccine wanes over time [1–5]. Longitudinal dynamics of the immune response following the administration of the second dose of the BNT162b2 vaccine revealed a substantial decrease at 6 months, regardless of the sex and age of the patients [6]. A systematic review and meta-regression [7] reported that the effectiveness of vaccines against COVID-19 has decreased by 2–30%. However, other studies have reported that the effectiveness of the vaccines in preventing severe disease shows a minimal decrease (9–10%) for up to 6 months [7–10]. The effectiveness of the vaccine against COVID-19-related hospitalization and death at ≥ 20 weeks after receiving two doses of the ChAdOx1-S or BNT162b2 vaccine has shown limited waning [11]. The effectiveness of the vaccine in preventing the incidence of COVID-19 must be considered while formulating a post-pandemic dosing plan; however, it is even more important to consider strategies for the prevention of severe disease [12]. Maintaining healthcare resources and preventing the overstraining of the healthcare system are critical for the mitigation of future pandemics.

The third (booster) dose is highly effective in preventing the incidence of severe disease [13–16]. The requirement for repeated booster doses has been discussed widely. Nevertheless, it is unclear whether further doses should be administered to individuals who have received the fourth or fifth dose during the pandemic or who have stopped after the second or third booster dose. The administration of booster doses to younger and low-risk populations has commenced; thus, the period during which the vaccine can maintain its effectiveness in terms of both the incidence and severity of COVID-19 needs to be established. Identifying the ideal timing for the administration of booster doses plays a crucial role in the formulation of public health policies and resource optimization.

Data regarding the waning of effectiveness of the vaccine is limited to a relatively short post-vaccination period or to age groups that do not include older adults or children or has been tracked to a limited extent following the emergence of the Omicron strain [14]. Therefore, this study investigated the duration of the effectiveness of the COVID-19 vaccine regarding the co-primary outcomes of the incidence of COVID-19 and COVID-19-related hospitalization. Real-world data for the population of a city in Japan comprising individuals of all ages were used in this study. Up to five doses of vaccinations had been administered during the Omicron wave in this population, and all individuals had been followed up for > 1 year after the administration of the third dose.

## Methods

### Data source and study design

Data were obtained from a linked database of healthcare administrative claims and vaccination records maintained by the municipality of a city in the Kanto region of Japan. The vaccine records were linked to health insurance claims data via unique identification numbers. The target population is all residents of the city who are covered by public insurance. All personal information was excluded, and de-identified data were sent to the researchers for secondary use.

The COVID-19 pandemic began in earnest at this time when the government declared a state of emergency on April 7, 2020, and began to restrict activities to ensure Social Distance. Therefore, April 1, 2020, which is also the start of the fiscal year, was chosen as the starting point for the study. On February 14, 2021, BNT162b2 COVID-19 vaccine was approved for production and marketing in Japan, and temporary inoculations based on the Immunization Law began on February 17 for healthcare workers, etc. Inoculations for the elderly, etc. began on April 12, and the target age for vaccination was changed from “16 years and older” to “12 years and older” on June 1.

According to the report by the Tokyo Metropolitan Government, the first wave was April-May 2020, the second wave was July-August 2020, the third wave was November-March 2020, the fourth wave was April-June 2021, and the fifth wave, when the alpha strain rapidly replaced the delta strain with high risk of serious illness The fifth wave of the Omicron strain, which has rapidly replaced the Delta strain with a higher risk of severe disease, began in July-October 2021 in which vaccination of the elderly has progressed, and the sixth wave of the Omicron strain began in January-May 2022, prompting the third round of vaccination. Seventh wave June 2022-. In Tokyo, the number of people who have completed the second dose of vaccination reached 70% of the total population in Tokyo in November 2021, and the vaccination rate reached approximately 80% as of May 2022.

The duration between April 1, 2020, and December 31, 2022, was set as the study period for this retrospective cohort study. The participants were already enrolled in the National Health Insurance system at the beginning of the study; therefore, their outcome data were considered to have been followed up from the beginning of the study to the point when they stopped receiving insurance coverage owing to death or relocation.

The healthcare administrative claims database comprises data regarding age and sex, as well as information regarding medical examinations and treatments that the individuals underwent on the dates of diagnosis for any disease during outpatient visits and hospital admissions. The International Classification of Diseases 10th revision (ICD-10) codes were used to identify the disease. The vaccination records included information on the types and dates of vaccination.

This study was approved by the institutional review board of the University of Tokyo. The requirement for obtaining informed consent was waived owing to the anonymized nature of the data.

### Study population

All individuals who were enrolled in the database during the study and baseline periods, which was defined as 1 year before the start date of the study period (April 1, 2020), were included in this study.

### Vaccination status

The vaccination status of the participants was defined as a time-dependent variable that distinguished the number of vaccinations. Individuals were included in the no-vaccination group from the commencement of the study until 14 days after receiving the first dose. Individuals who did not receive any doses of the vaccine during the study period were included in the no-vaccine group throughout the study period. The time point for the one-dose vaccine group was from 14 days after the first dose to 14 days after the second dose. The time points for the two-, three-, four-, or five-dose vaccine groups were also set similarly. The groups based on the total number of vaccinations were included in the descriptive analysis.

### Outcomes and covariates

The time to the first incidence of COVID-19 determined from the beginning of the study period, which was confirmed using the ICD-10 code U071 without a suspected disease flag, was defined as the first co-primary outcome. The time to the first COVID-19 hospitalization determined from the beginning of the study period, which was confirmed using the ICD-10 code U071 without a suspected disease flag, was defined as the second co-primary outcome.

Age (continuous variable) at the beginning of the study period, sex, and comorbidities (including cancer [C0-97], diabetes [E10-14], chronic obstructive pulmonary disease [J440-441, J448-449], asthma [J45], chronic kidney disease [N18], cardiovascular disease [I1, I5-13, I20-25, I27, I30-51, I60-69], obesity [E66], and hypertension [I10] confirmed at the baseline period and without suspected disease flag) were used as covariates in the adjusted analysis of the effectiveness of the vaccine.

### Statistical analyses

The characteristics of the participants were stratified according to the total number of vaccinations. A time-dependent Cox proportional hazard model [17] with time-dependent vaccine status only (univariable analysis) and covariates confirmed in the baseline period (multivariable analysis) was used to estimate the effectiveness of one, two, three, four, and five doses of the COVID-19 vaccine on the two co-primary outcomes. The probability of the incidence of COVID-19 and COVID-19-related hospitalization, estimated via univariable analysis using the Breslow estimator [18], was plotted. Subgroup analyses were conducted based on age categories (0–9, 10–19, 20–29, 30–39, 40–49, 50–59, 60–69, 70–79, 80–89, and ≥ 90 years) for overall effectiveness of the COVID-19 vaccine comparing the non-vaccine group vs. the overall vaccine group including the groups with all doses. A time-dependent piecewise Cox proportional hazard model with the time-dependent vaccine status and time (14 days–1 month, 1–2 months, 2–3 months, 3–4 months, 4–5 months, 5–6 months, 6–7 months, 7–8 months, 8–9 months, 9–10 months, 10–11 months, 11–12 months, and ≥ 12 months after the COVID-19 vaccination) serving as interaction terms and covariates confirmed in the baseline period was used to investigate the time-dependent effectiveness of the COVID-19 vaccination against the co-primary outcomes. All participants were included in the at-risk population based on the calendar time according to their follow-up period to account for the confounding effect of the differences in COVID-19 epidemics and strains. All statistical analyses were performed using SAS ver. 9.4.

## Results

**Fig. 1:**
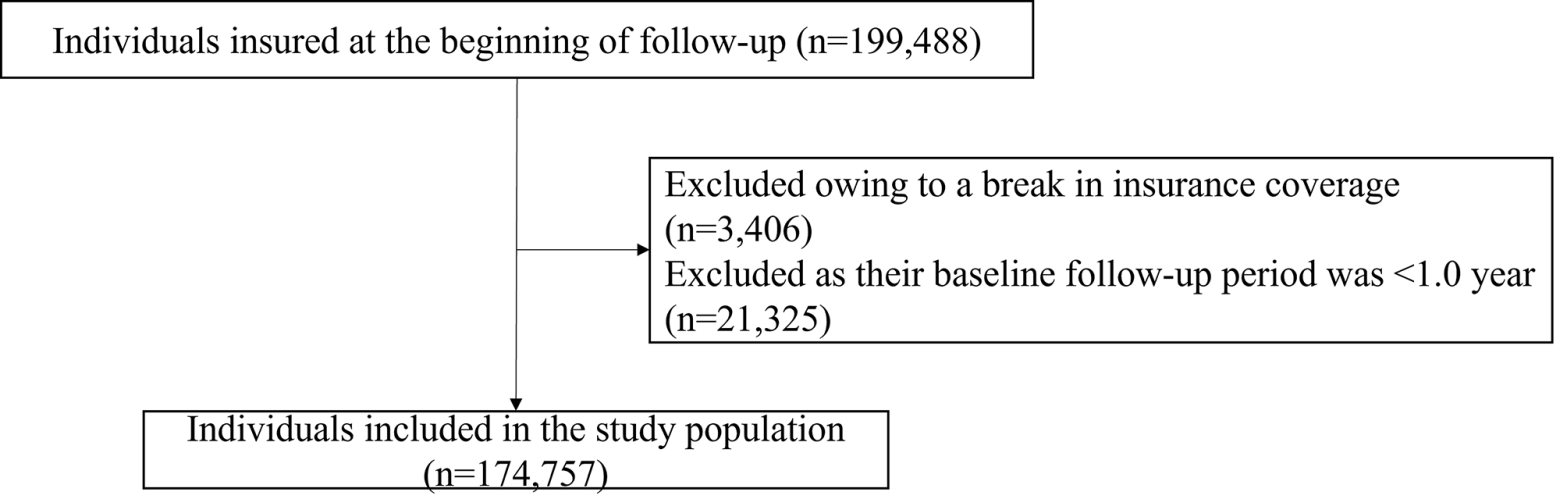
Selection of participants to analyze the effectiveness of the COVID-19 vaccine in preventing the incidence of COVID-19 or hospitalization. COVID-19: Coronavirus disease 2019

Among the 199,488 individuals who were insured at the beginning of the study period from April 1, 2020, to December 31, 2022 (Figs. 1), 3,406 individuals without a continuous history of health insurance coverage and 21,325 participants who were not insured during the baseline period of 1.0 year were excluded. Thus, 174,757 individuals were included in the analysis population. A total of 14,416 individuals (8.3%) were diagnosed with COVID-19 and 936 (0.54%) were hospitalized for COVID-19 during the study period.

Table 1 presents the baseline characteristics of the participants stratified according to the total number of vaccinations received. The mean age and proportion of women increased as the total number of vaccine doses increased. Table 2 presents the results of the univariable and multivariable analyses using the time-dependent Cox regression model with reference of non-vaccine group. Multivariable analysis revealed a lower incidence of COVID-19 in the one-dose group (hazard ratio, 0.76 [95% confidence interval, 0.63–0.91]), two-dose (0.89 [0.85–0.93]), three-dose (0.80 [0.76–0.85]), four-dose (0.93 [0.88–1.00]), and five-dose (0.72 [0.62–0.84]) groups compared with that in the no-vaccine group. Similarly, a lower incidence of COVID-19-related hospitalization was observed in the one-dose (0.42 [0.21–0.81]), two-dose (0.44 [0.35–0.56]), three-dose (0.38 [0.30–0.47]), four-dose (0.20 [0.14–0.28]), and five-dose (0.11 [0.014–0.86]) groups compared with that in the no-vaccine group. Supplemental Table 1 presents the results with the exchanged reference of the standard vaccine scheme (two or three dose). Additional dose against the standard two or three doses may not gain the benefit in terms of prevention of infection. On the other hand, in terms of prevention of hospitalization, in other words severity of illness, it was suggested that one may have benefit from additional doses against the standard vaccine scheme (two or three doses).

**Table 1:** Baseline characteristics of the study population

Characteristics	All individuals (*n* = 174,757)	No vaccine (*n* = 37,637)	One dose of vaccine (*n* = 953)	Two doses of vaccine (*n* = 17,340)	Three doses of vaccine (*n* = 22,146)	Four doses of vaccine (*n* = 28,172)	Five doses of vaccine (*n* = 68,509)	*P*-value^*^
Age (years)ーmean ± standard deviation	62.8 ± 21.9	51.8 ± 28.3	58.9 ± 26.2	49.1 ± 24.3	54.5 ± 22.0	63.9 ± 17.7	74.6 ± 7.9	< 0.001
Age category (years)ーno. (%)								< 0.001
0–9	4,643 (2.7)	4,110 (10.9)	34 (3.6)	329 (1.9)	166 (0.7)	4 (0.0)	0 (0)	
10–19	5,910 (3.4)	1,731 (4.6)	62 (6.5)	1,877 (10.8)	1,634 (7.4)	599 (2.1)	7 (0.0)	
20–29	8,500 (4.9)	4,085 (10.9)	88 (9.2)	2,090 (12.1)	1,573 (7.1)	619 (2.2)	45 (0.1)	
30–39	10,190 (5.8)	3,831 (10.2)	71 (7.5)	2,574 (14.8)	2,289 (10.3)	1,329 (4.7)	96 (0.1)	
40–49	13,884 (7.9)	3,828 (10.2)	94 (9.9)	2,713 (15.6)	3,545 (16.0)	3,418 (12.1)	286 (0.4)	
50–59	14,957 (8.6)	3,377 (9.0)	73 (7.7)	1,787 (10.3)	3,465 (15.6)	4,820 (17.1)	1,435 (2.1)	
60–69	25,895 (14.8)	3,157 (8.4)	83 (8.7)	1,266 (7.3)	2,613 (11.8)	4,488 (15.9)	14,288 (20.9)	
70–79	52,355 (30.0)	5,158 (13.7)	170 (17.8)	1,972 (11.4)	3,461 (15.6)	7,124 (25.3)	34,470 (50.3)	
80–89	32,295 (18.5)	5,852 (15.5)	210 (22.0)	2,148 (12.4)	2,828 (12.8)	4,960 (17.6)	16,297 (23.8)	
≥ 90	6,128 (3.5)	2,508 (6.7)	68 (7.1)	584 (3.4)	572 (2.6)	811 (2.9)	1,585 (2.3)	
Male sexーno. (%)	82,279 (47.1)	20,174 (53.6)	497 (52.2)	9,289 (53.6)	10,624 (48.0)	12,549 (44.5)	29,146 (42.5)	< 0.001
Clinical risk factorsーno. (%)								
Cancer	781 (0.4)	147 (0.4)	3 (0.3)	42 (0.2)	62 (0.3)	120 (0.4)	407 (0.6)	< 0.001
Diabetes	2,909 (1.7)	436 (1.2)	17 (1.8)	220 (1.3)	322 (1.5)	493 (1.7)	1,421 (2.1)	< 0.001
Chronic obstructive pulmonary disease	516 (0.3)	81 (0.2)	9 (0.9)	33 (0.2)	57 (0.3)	69 (0.2)	267 (0.4)	< 0.001
Asthma	4,531 (2.6)	1,166 (3.1)	32 (3.4)	473 (2.7)	587 (2.7)	650 (2.3)	1,623 (2.4)	< 0.001
Chronic kidney disease	906 (0.5)	175 (0.5)	7 (0.7)	57 (0.3)	93 (0.4)	142 (0.5)	432 (0.6)	< 0.001
Cardiovascular disease	1,540 (0.9)	268 (0.7)	13 (1.4)	90 (0.5)	135 (0.6)	249 (0.9)	785 (1.1)	< 0.001
Obesity	129 (0.1)	29 (0.1)	0 (0)	10 (0.1)	25 (0.1)	24 (0.1)	41 (0.1)	0.143
Hypertension	3,004 (1.7)	400 (1.1)	10 (0.1)	219 (1.3)	337 (1.5)	555 (2.0)	1,483 (2.2)	< 0.001

*F-test for continuous age, chi-squared test for binomial or ordinal categorical variables

COVID-19: Coronavirus disease 2019

**Table 2:** Time-dependent Cox regression analysis for the incidence of COVID-19 and COVID-19-related hospitalization with reference of non-vaccine group

	Incidence of COVID-19			Hospitalization of COVID-19	
	Hazard ratio (95% confidence interval)	*P*-value		Hazard ratio (95% confidence interval)	*P*-value
*Univariable analysis*					
One-dose COVID-19 vaccine group	0.68 (0.57, 0.81)	< 0.001		0.51 (0.26, 0.98)	0.045
Two-dose COVID-19 vaccine group	0.78 (0.74, 0.82)	< 0.001		0.54 (0.43, 0.68)	< 0.001
Three-dose COVID-19 vaccine group	0.57 (0.54, 0.59)	< 0.001		0.58 (0.46, 0.73)	< 0.001
Four-dose COVID-19 vaccine group	0.55 (0.52, 0.58)	< 0.001		0.37 (0.26, 0.53)	< 0.001
Five-dose COVID-19 vaccine group	0.41 (0.35, 0.47)	< 0.001		0.21 (0.027, 1.66)	0.14
*Multivariable analysis*					
One-dose COVID-19 vaccine group	0.76 (0.63, 0.91)	0.003		0.42 (0.21, 0.81)	0.01
Two-dose COVID-19 vaccine group	0.89 (0.85, 0.93)	< 0.001		0.44 (0.35, 0.56)	< 0.001
Three-dose COVID-19 vaccine group	0.80 (0.76, 0.85)	< 0.001		0.38 (0.30, 0.47)	< 0.001
Four-dose COVID-19 vaccine group	0.93 (0.88, 0.99)	0.033		0.20 (0.14, 0.28)	< 0.001
Five-dose COVID-19 vaccine group	0.72 (0.62, 0.84)	< 0.001		0.11 (0.014, 0.86)	0.036
Age (years)	0.98 (0.98, 0.98)	< 0.001		1.04 (1.03, 1.04)	< 0.001
Female sex	1.04 (1.01, 1.08)	0.019		0.66 (0.58, 0.75)	< 0.001
Clinical risk factors					
Cancer	1.18 (0.91, 1.52)	0.211		1.19 (0.53, 2.66)	0.67
Diabetes	0.94 (0.82, 1.08)	0.364		0.92 (0.56, 1.51)	0.75
Chronic obstructive pulmonary disease	1.24 (0.91, 1.68)	0.18		0.86 (0.28, 2.68)	0.8
Asthma	1.30 (1.19, 1.42)	< 0.001		1.16 (0.76, 1.77)	0.49
Chronic kidney disease	1.41 (1.12, 1.77)	0.003		1.43 (0.74, 2.77)	0.28
Cardiovascular disease	1.32 (1.11, 1.57)	0.002		1.03 (0.55, 1.93)	0.92
Obesity	1.30 (0.80, 2.13)	0.293		-	-
Hypertension	1.04 (0.92, 1.19)	0.531		1.05 (0.66, 1.68)	0.83

COVID-19: Coronavirus disease 2019

Obesity was removed from the model for hospitalization due to the diverged estimate such as hazard ratio = 0

Table 3 presents the results of the subgroup analyses performed according to the age categories (0–9, 19–19, 20–29, 30–39, 40–49, 50–59, 60–69, 70–79, 80–89, and ≥ 90 years) for the overall effectiveness of the COVID-19 vaccine based on time-dependent univariable Cox regression analysis with reference of non-vaccine group. Subgroup analyses revealed that the incidence of COVID-19 was inconsistent. The effectiveness against COVID-19-related hospitalization was significant among older individuals except among those aged ≥ 90 years.

**Table 3:** Subgroup analysis according to the age categories for overall effectiveness of the COVID-19 vaccine in the no-vaccine group (reference) vs. vaccine group including all dose groups based on time-dependent univariable Cox regression analysis for the incidence of COVID-19 and COVID-19-related hospitalization

	Incidence of COVID-19			COVID-19-related hospitalization	
	Hazard ratio (95% confidence interval)	*P*-value		Hazard ratio (95% confidence interval)	*P*-value
*Overall effectiveness of the COVID-19 vaccine in non-vaccine group (reference) vs. vaccine group including all dose groups*					
	0.62 (0.60, 0.65)	< 0.001		0.52 (0.44, 0.63)	< 0.001
*Subgroup analysis based on age (years)*					
0–9	0.63 (0.45, 0.89)	0.008		- (-, -)	-
10–19	0.77 (0.67, 0.87)	< 0.001		- (-, -)	-
20–29	0.80 (0.68, 0.93)	0.005		0.23 (0.022, 2.42)	0.22
30–39	1.03 (0.90, 1.18)	0.64		0.57 (0.11, 2.91)	0.50
40–49	1.08 (0.95, 1.23)	0.24		0.18 (0.056, 0.59)	0.004
50–59	1.16 (1.00, 1.34)	0.050		0.20 (0.096, 0.42)	< 0.001
60–69	0.85 (0.73, 0.99)	0.038		0.19 (0.11, 0.32)	< 0.001
70–79	0.94 (0.82, 1.08)	0.41		0.27 (0.18, 0.38)	< 0.001
80–89	1.29 (1.11, 1.50)	< 0.001		0.56 (0.40, 0.79)	< 0.001
≥ 90	3.07 (2.29, 4.12)	< 0.001		1.59 (0.82, 3.10)	0.17

COVID-19: Coronavirus disease 2019

**Fig. 2:**
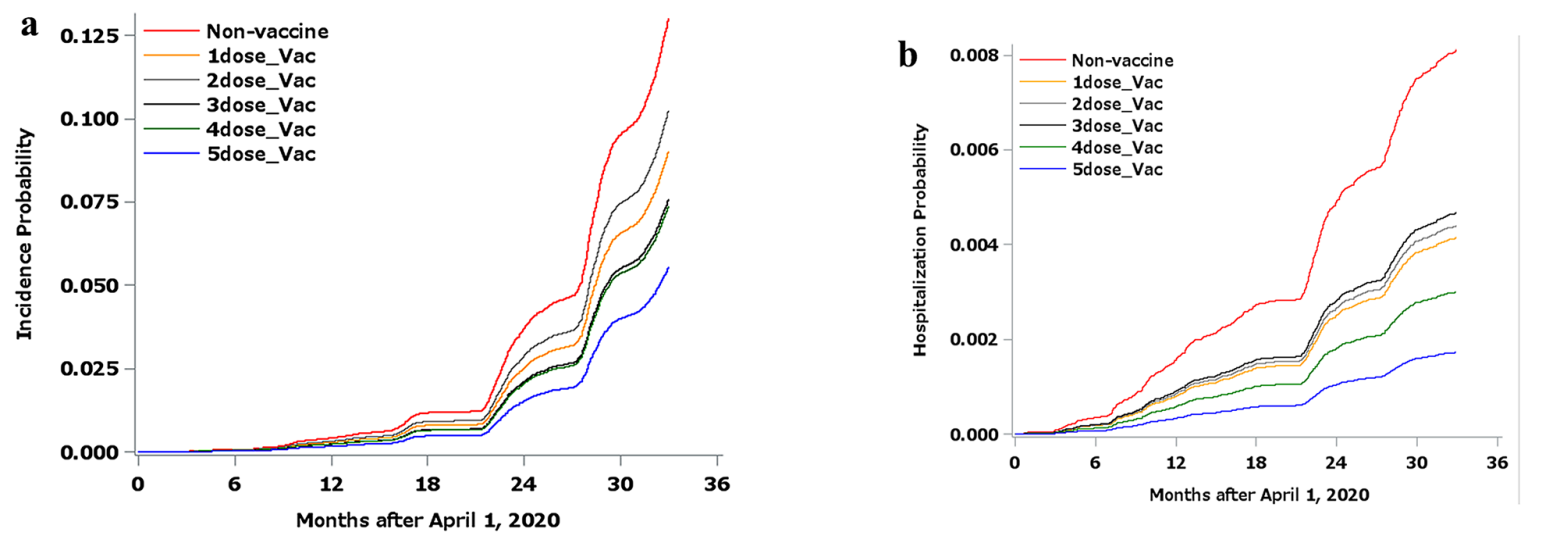
**a**) Probability of the incidence of COVID-19 estimated by the Breslow estimator in univariate analysis using a time-dependent Cox proportional hazard model with time-dependent vaccine status only. Note that this is different from the occurrence curve in the actual population. COVID-19: Coronavirus disease 2019. **b**) Incidence probability of the requirement for hospitalization for COVID-19 estimated by the Breslow estimator in univariate analysis using a time-dependent Cox proportional hazard model with time-dependent vaccine status only. Note that this is different from the occurrence curve in the actual population. COVID-19: Coronavirus disease 2019

Figure 2a and 2b present the probability of the outcomes estimated by the Breslow estimator in the univariable analyses. Note that this is different from the occurrence curve in the actual population. The estimated probability of the incidence of COVID-19 was lower in the vaccinated groups. The incidence of COVID-19 generally decreased as the frequency of vaccination increased (five-dose vs. three- or four-dose vs. one- or two-dose groups). The estimated probability of COVID-19-related hospitalization was lower in the vaccinated groups. The incidence of COVID-19-related hospitalization generally decreased as the frequency of vaccination increased (four- or five-dose vs. one-, two-, or three-dose groups).

**Fig. 3:**
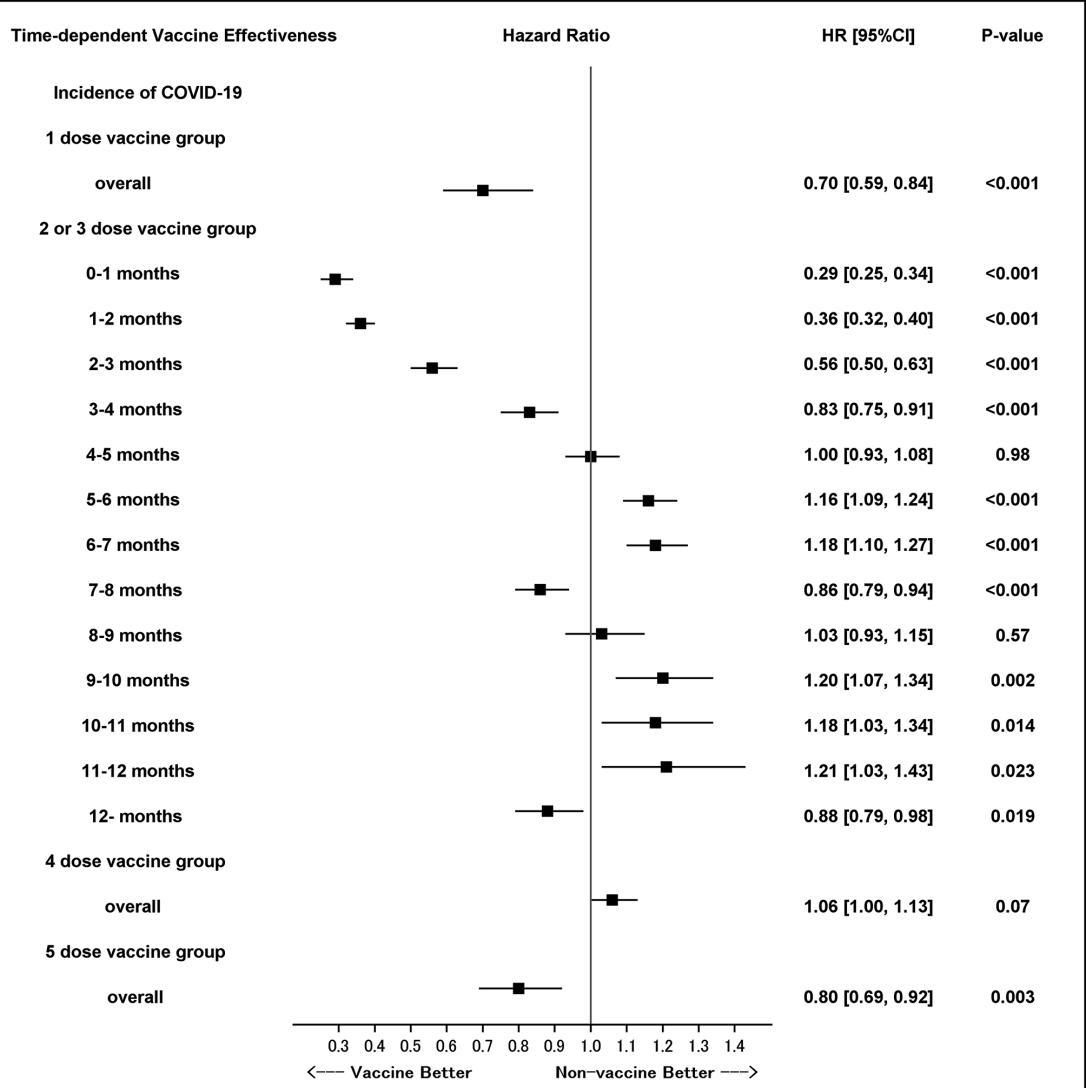
Duration of the effectiveness of the COVID-19 vaccine in preventing the incidence of COVID-19 after vaccination with reference of non-vaccine group. Time-dependent piecewise Cox proportional hazard model with the time-dependent vaccine status and time (14 days–1 month, 1–2 months, 2–3 months, 3–4 months, 4–5 months, 5–6 months, 6–7 months, 7–8 months, 8–9 months, 9–10 months, 10–11 months, 11–12 months, and ≥ 12 months after the COVID-19 vaccination) serving as interaction terms, and covariates confirmed in the baseline period, namely, age, sex, and history of cancer, diabetes, chronic obstructive pulmonary disease, asthma, chronic kidney disease, and cardiovascular disease. COVID-19: Coronavirus disease 2019

Figure 3 presents the effectiveness of the vaccine against the incidence of COVID-19 according to the multivariable analysis based on the time-dependent piecewise Cox proportional hazard model with reference of non-vaccine group. The effectiveness of two and three doses waned by approximately 4 months. The point estimate of the hazard ratio ranged from 0.29 to 0.83 by 4 months and from 0.86 to 1.21 thereafter. The preventive effects showed non-linear time-dependency and were statistically significant at 0–1, 1–2, 2–3, 3–4, 7–8, and ≥ 12 months. Supplemental Table 1 presents the time dependency of the effectiveness of the vaccine for all dose groups in the univariable analysis.

**Fig. 4:**
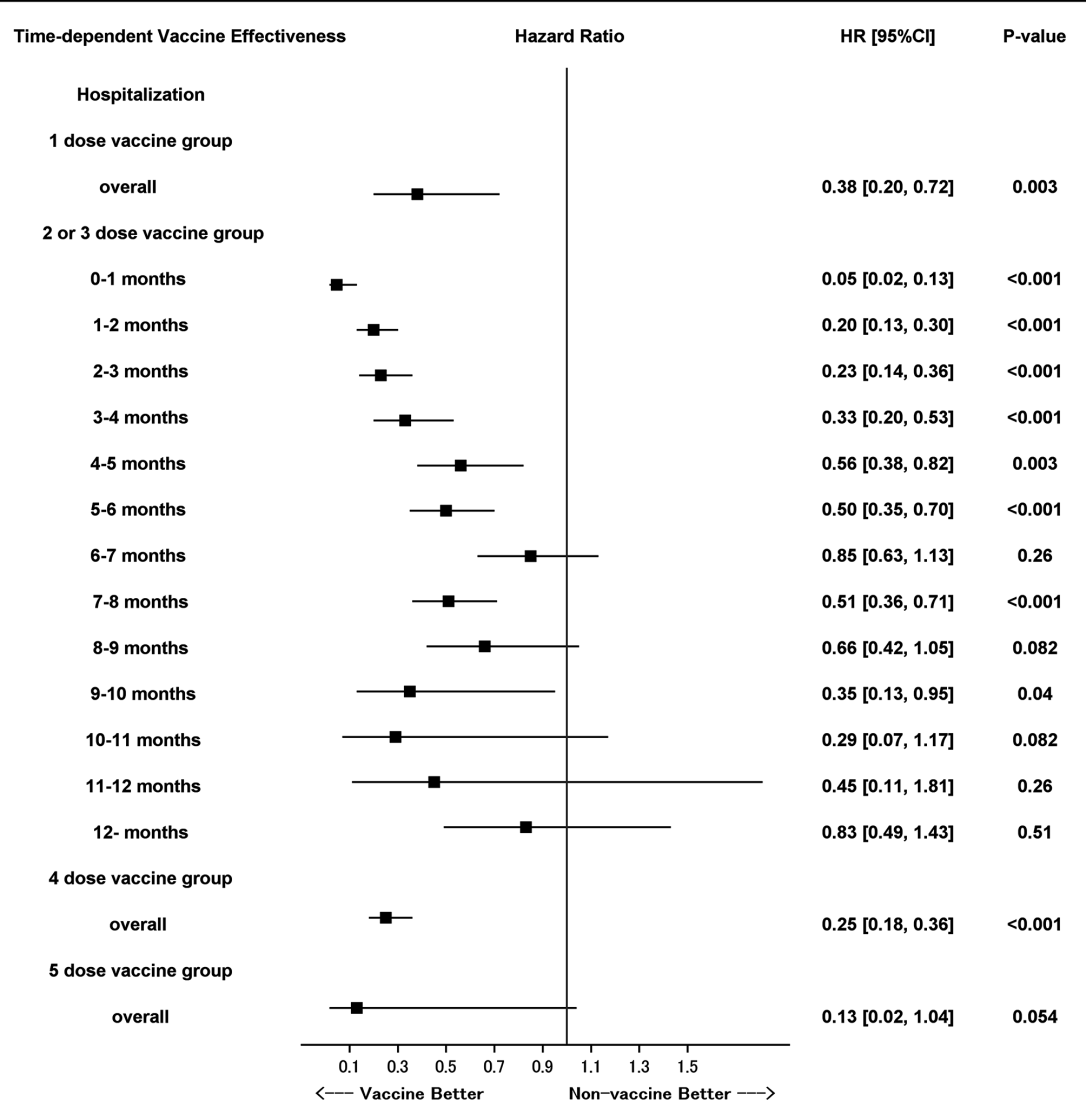
Duration of the effectiveness of the COVID-19 vaccine in preventing COVID-19-related hospitalization after vaccination with reference of non-vaccine group. Time-dependent piecewise Cox proportional hazard model with the time-dependent vaccine status and time (14 days–1 month, 1–2 months, 2–3 months, 3–4 months, 4–5 months, 5–6 months, 6–7 months, 7–8 months, 8–9 months, 9–10 months, 10–11 months, 11–12 months, and ≥ 12 months after the COVID-19 vaccination) serving as interaction terms, and covariates confirmed in the baseline period, namely, age, sex, and history of cancer, diabetes, chronic obstructive pulmonary disease, asthma, chronic kidney disease, and cardiovascular disease. COVID-19: Coronavirus disease 2019

Figure 4 presents the effectiveness of the vaccine in preventing COVID-19-related hospitalization according to multivariable analysis based on the time-dependent piecewise Cox proportional hazard model with reference of non-vaccine group. The point estimates of the hazard ratio for two or three doses gradually increased in later months; however, they were < 1 throughout the observation period. The preventive effects showed non-linear time-dependency and were significant at 0–1, 1–2, 2–3, 3–4, 4–5, 5–6, 7–8, and 9–10 months. Supplemental Table 2 presents the time dependency of the effectiveness of the vaccine for all dose groups in the univariable analysis.

## Discussion

A population-based retrospective cohort study was conducted using a linked database of healthcare administrative claims data and vaccination records of a city in Japan for a period extending from April 1, 2020, to December 31, 2022. Assessment of the effectiveness of one, two, three, four, and five doses of the COVID-19 vaccine on the two co-primary outcomes revealed the more definitive effectiveness against COVID-19-related hospitalization than against the incidence of COVID-19 in each dose group. The effectiveness of the vaccine against severe disease became more evident as the number of booster doses administered increased, which may be attributed to the recovery of effectiveness owing to the increase in antibody titers. Consistent with the findings of previous studies, the findings of the present study underscore the critical role of continuous and periodic booster vaccinations, especially in reducing the severity of COVID-19, even amidst the emergence of new variants, such as Omicron JN.1.

The duration of effectiveness of the COVID-19 vaccine in preventing the incidence of COVID-19 and COVID-19-related hospitalization was investigated in this study. The effectiveness of the vaccine showed gradual attenuation with time after vaccination and the protective effectiveness was maintained for 4 months for the incidence of COVID-19 and ≥ 6 months for COVID-19-related hospitalization. The fifth wave of the Omicron strain began in July-October 2021 in which vaccination of the elderly has progressed with the second dose, and the sixth wave of the Omicron strain began in January-May 2022, prompting the third dose. The fact that the vaccine was not yet available for the Omicron strain at this time may have contributed to the lack of significant effectiveness for the incidence ≥ 5 months.

The waning of the protective effectiveness against COVID-19-related hospitalization was milder than that against incidence. These findings are consistent with the trends reported by existing studies on the attenuation of the effectiveness of the first vaccine without booster dose and the second or third booster dose over time [7–16]. Furthermore, the present study indicated that the effectiveness against severe disease after the second or third booster dose persisted for > 6 months. The trends shown in Fig. 3 suggest that the effectiveness persisted for up to 1 year. Vaccinating younger individuals and individuals at low risk of severe disease every year, instead of every 6 months, may be sufficient. The findings of the present study may be useful for formulating routine vaccination plans after a pandemic. In addition, the duration of effectiveness against hospitalization obtained in this study and the methods used to study it will be helpful in making policy decisions regarding the content of vaccination campaigns to control the total number of severe cases below a certain level in each regional population, rather than simply prioritizing the elderly and high-risk individuals for vaccination, even in a future pandemic, as the whole world has experienced the importance of avoiding the worst case scenario of a breakdown of medical resources and systems. The present study has a few limitations. First, although the primary outcome was the incidence of COVID-19, the data did not include information on the results of COVID-19 antigen or polymerase chain reaction tests. Second, there may have been unmeasured confounding factors. Individuals who received a greater number of booster doses may have been biased toward individuals who were more likely to be infected or hospitalized. Third, the duration of the study was until December 31, 2022; thus, the follow-up period after the fourth and fifth doses was insufficient. Fourth, it has not been investigated whether different types of vaccines have different effectiveness and whether they affect the waning of the protective effectiveness. Fifth, we were unable to consider strain cross-reactivity, dose cross-reactivity, and personal immune conditions with regard to COVID-19.

## Conclusion

The present study identified a more definite effectiveness of the vaccine against COVID-19-related hospitalization, regardless of the number of booster doses administered; the effectiveness against the incidence of COVID-19 was not as strong. Vaccine effectiveness showed a gradual attenuation with time after vaccination and maintained effectiveness against the incidence of COVID-19 for 4 months and ≥ 6 months for COVID-19-related hospitalization. These findings will aid in identifying the optimal timing for the formulation of a routine vaccination plan after a pandemic.

### Electronic Supplementary Material

Below is the link to the electronic supplementary material.

Supplementary Material 1

## Data Availability

The database used in this study is maintained by a city in the Kanto region of Japan. Restrictions applied to the availability of data were applied with permission for this study. Accordingly, these data are not publicly available.

## Abbreviations

CI: Confidence Interval
COVID-19: Coronavirus Disease 2019
HR: Hazard Ratio
ICD-10: International Classification of Diseases, 10th revision
