# Supplementary material for: Duration of effectiveness of the COVID-19 vaccine in Japan: a retrospective cohort study using large-scale population-based registry data
Source: BMC Infect Dis. 2024 Jun 28;24:648. doi: 10.1186/s12879-024-09488-6 (PMC11212202; doi:10.1186/s12879-024-09488-6)
Supplement: Supplementary file 1 — Supplementary Material 1 [file 12879_2024_9488_MOESM1_ESM.docx]

**Supplemental Table 1.** Time-dependent Cox regression analysis for the incidence of COVID-19 and COVID-19-related hospitalization with reference as two or three-dose COVID-19 vaccine group

|  | Incidence of COVID-19 | |  | Hospitalization of COVID-19 | |
| --- | --- | --- | --- | --- | --- |
|  | Hazard ratio  (95% confidence interval) | P-value |  | Hazard ratio  (95% confidence interval) | P-value |
| *Univariable analysis* |  |  |  |  |  |
| No vaccine group | 1.53 (1.47, 1.6) | < 0.001 |  | 1.8 (1.49, 2.17) | < 0.001 |
| One-dose COVID-19 vaccine group | 1.01 (0.84, 1.21) | 0.95 |  | 0.92 (0.47, 1.79) | 0.81 |
| Four-dose COVID-19 vaccine group | 0.87 (0.82, 0.91) | < 0.001 |  | 0.66 (0.47, 0.93) | 0.017 |
| Five-dose COVID-19 vaccine group | 0.64 (0.55, 0.73) | < 0.001 |  | 0.38 (0.049, 2.95) | 0.35 |
| *Multivariable analysis* |  |  |  |  |  |
| No vaccine group | 1.18 (1.13, 1.23) | < 0.001 |  | 2.46 (2.03, 2.97) | < 0.001 |
| One-dose COVID-19 vaccine group | 0.89 (0.74, 1.07) | 0.21 |  | 1.0 (0.51, 1.97) | 0.99 |
| Four-dose COVID-19 vaccine group | 1.12 (1.07, 1.19) | < 0.001 |  | 0.51 (0.35, 0.71) | < 0.001 |
| Five-dose COVID-19 vaccine group | 0.87 (0.75, 1.0) | 0.05 |  | 0.28 (0.035, 2.15) | 0.22 |

COVID-19: Coronavirus disease 2019

**Supplemental Table 2.** Time-dependent effectiveness of the COVID-19 vaccine against the incidence of COVID-19 based on time-dependent piecewise univariable Cox regression analysis

|  |  | Hazard ratio | 95% confidence interval | | | | | P-value |
| --- | --- | --- | --- | --- | --- | --- | --- | --- |
| One-dose COVID-19 vaccine group | Overall | 0.56 | ( | 0.47 | , | 0.68 | ) | < 0.001 |
| Two-dose COVID-19 vaccine group | 14 days–1 month | 0.15 | ( | 0.11 | , | 0.23 | ) | < 0.001 |
|  | 1 month–2 months | 0.14 | ( | 0.11 | , | 0.18 | ) | < 0.001 |
|  | 2 months–3 months | 0.30 | ( | 0.24 | , | 0.37 | ) | < 0.001 |
|  | 3 months–4 months | 1.05 | ( | 0.91 | , | 1.22 | ) | 0.50 |
|  | 4 months–5 months | 1.22 | ( | 1.08 | , | 1.38 | ) | 0.001 |
|  | 5 months–6 months | 1.08 | ( | 0.96 | , | 1.21 | ) | 0.18 |
|  | 6 months–7 months | 0.84 | ( | 0.76 | , | 0.93 | ) | 0.001 |
|  | 7 months–8 months | 0.54 | ( | 0.49 | , | 0.60 | ) | < .001 |
|  | 8 months–9 months | 0.77 | ( | 0.67 | , | 0.88 | ) | < 0.001 |
|  | 9 months–10 months | 1.22 | ( | 1.06 | , | 1.40 | ) | 0.005 |
|  | 10 months–11 months | 1.22 | ( | 1.06 | , | 1.41 | ) | 0.006 |
|  | 11 months–12 months | 1.19 | ( | 1.00 | , | 1.40 | ) | 0.048 |
|  | ≥ 12 months | 0.78 | ( | 0.70 | , | 0.87 | ) | < 0.001 |
| Three-dose Covid-19 vaccine group | 14 days–1 month | 0.21 | ( | 0.17 | , | 0.25 | ) | < 0.001 |
|  | 1 month–2 months | 0.29 | ( | 0.25 | , | 0.33 | ) | < 0.001 |
|  | 2 months–3 months | 0.45 | ( | 0.39 | , | 0.51 | ) | < 0.001 |
|  | 3 months–4 months | 0.56 | ( | 0.50 | , | 0.64 | ) | < 0.001 |
|  | 4 months–5 months | 0.67 | ( | 0.61 | , | 0.74 | ) | < 0.001 |
|  | 5 months–6 months | 0.75 | ( | 0.70 | , | 0.81 | ) | < 0.001 |
|  | 6 months–7 months | 0.91 | ( | 0.82 | , | 1.00 | ) | 0.049 |
|  | 7 months–8 months | 0.99 | ( | 0.85 | , | 1.15 | ) | 0.85 |
|  | 8 months–9 months | 0.93 | ( | 0.79 | , | 1.11 | ) | 0.42 |
|  | 9 months–10 months | 0.89 | ( | 0.73 | , | 1.07 | ) | 0.21 |
|  | 10 months–11 months | 0.80 | ( | 0.60 | , | 1.07 | ) | 0.13 |
|  | 11 months–12 months | 0.83 | ( | 0.34 | , | 2.00 | ) | 0.68 |
|  | ≥ 12 months | - | ( | - | , | - | ) | - |
| Four-dose COVID-19 vaccine group | 14 days–1 month | 0.46 | ( | 0.41 | , | 0.52 | ) | < 0.001 |
|  | 1 month–2 months | 0.54 | ( | 0.49 | , | 0.59 | ) | < 0.001 |
|  | 2 months–3 months | 0.62 | ( | 0.55 | , | 0.69 | ) | < 0.001 |
|  | 3 months–4 months | 0.80 | ( | 0.72 | , | 0.89 | ) | < 0.001 |
|  | 4 months–5 months | 0.93 | ( | 0.84 | , | 1.03 | ) | 0.14 |
|  | 5 months–6 months | 1.02 | ( | 0.87 | , | 1.20 | ) | 0.79 |
|  | 6 months–7 months | 0.79 | ( | 0.33 | , | 1.91 | ) | 0.60 |
|  | 7 months–8 months | - | ( | - | , | - | ) | - |
|  | 8 months–9 months | - | ( | - | , | - | ) | - |
|  | 9 months–10 months | - | ( | - | , | - | ) | - |
|  | 10 months–11 months | - | ( | - | , | - | ) | - |
|  | 11 months–12 months | - | ( | - | , | - | ) | - |
|  | ≥ 12 months | - | ( | - | , | - | ) | - |
| Five-dose COVID-19 vaccine group | 14 days–1 month | 0.52 | ( | 0.44 | , | 0.62 | ) | < 0.001 |
|  | 1 month–2 months | 0.52 | ( | 0.41 | , | 0.66 | ) | < 0.001 |
|  | 2 months–3 months | - | ( | - | , | - | ) | - |
|  | 3 months–4 months | - | ( | - | , | - | ) | - |
|  | 4 months–5 months | - | ( | - | , | - | ) | - |
|  | 5 months–6 months | - | ( | - | , | - | ) | - |
|  | 6 months–7 months | - | ( | - | , | - | ) | - |
|  | 7 months–8 months | - | ( | - | , | - | ) | - |
|  | 8 months–9 months | - | ( | - | , | - | ) | - |
|  | 9 months–10 months | - | ( | - | , | - | ) | - |
|  | 10 months–11 months | - | ( | - | , | - | ) | - |
|  | 11 months–12 months | - | ( | - | , | - | ) | - |
|  | ≥ 12 months | - | ( | - | , | - | ) | - |

**Supplemental Table 3.** Time-dependent effectiveness of the vaccine against hospitalization for COVID-19 based on time-dependent piecewise univariable Cox regression analysis

|  |  | Hazard ratio | 95% confidence interval | | | | | P value |
| --- | --- | --- | --- | --- | --- | --- | --- | --- |
| One-dose COVID-19 vaccine group | Overall | 0.46 | ( | 0.24 | , | 0.86 | ) | 0.015 |
| Two-dose COVID-19 vaccine group | 14 days–1 month | - | ( | - | , | - | ) | 0.94 |
|  | 1 month–2 months | 0.14 | ( | 0.06 | , | 0.35 | ) | < 0.001 |
|  | 2 months–3 months | 0.22 | ( | 0.10 | , | 0.48 | ) | < 0.001 |
|  | 3 months–4 months | 0.26 | ( | 0.12 | , | 0.58 | ) | < 0.001 |
|  | 4 months–5 months | 0.42 | ( | 0.22 | , | 0.80 | ) | 0.008 |
|  | 5 months–6 months | 0.38 | ( | 0.20 | , | 0.71 | ) | 0.002 |
|  | 6 months–7 months | 0.82 | ( | 0.57 | , | 1.17 | ) | 0.27 |
|  | 7 months–8 months | 0.67 | ( | 0.46 | , | 0.95 | ) | 0.026 |
|  | 8 months–9 months | 0.99 | ( | 0.60 | , | 1.62 | ) | 0.95 |
|  | 9 months–10 months | 0.40 | ( | 0.13 | , | 1.26 | ) | 0.12 |
|  | 10 months–11 months | - | ( | - | , | - | ) | 0.95 |
|  | 11 months–12 months | 0.47 | ( | 0.11 | , | 1.92 | ) | 0.29 |
|  | ≥ 12 months | 1.59 | ( | 0.90 | , | 2.81 | ) | 0.11 |
| Three-dose COVID-19 vaccine group | 14 days–1 month | 0.10 | ( | 0.04 | , | 0.26 | ) | < 0.001 |
|  | 1 month–2 months | 0.42 | ( | 0.26 | , | 0.68 | ) | < 0.001 |
|  | 2 months–3 months | 0.46 | ( | 0.26 | , | 0.81 | ) | 0.007 |
|  | 3 months–4 months | 0.56 | ( | 0.31 | , | 1.00 | ) | 0.051 |
|  | 4 months–5 months | 0.85 | ( | 0.52 | , | 1.37 | ) | 0.5 |
|  | 5 months–6 months | 0.88 | ( | 0.58 | , | 1.35 | ) | 0.57 |
|  | 6 months–7 months | 2.09 | ( | 1.33 | , | 3.27 | ) | 0.001 |
|  | 7 months–8 months | 1.40 | ( | 0.59 | , | 3.35 | ) | 0.45 |
|  | 8 months–9 months | 1.52 | ( | 0.45 | , | 5.15 | ) | 0.5 |
|  | 9 months–10 months | 0.95 | ( | 0.13 | , | 7.28 | ) | 0.96 |
|  | 10 months–11 months | 6.13 | ( | 1.30 | , | 28.99 | ) | 0.022 |
|  | 11 months–12 months | - | ( | - | , | - | ) | 1.00 |
|  | ≥ 12 months | - | ( | - | , | - | ) | 1.00 |
| Four-dose COVID-19 vaccine group | 14 days–1 month | 0.33 | ( | 0.15 | , | 0.73 | ) | 0.006 |
|  | 1 month–2 months | 0.46 | ( | 0.25 | , | 0.85 | ) | 0.013 |
|  | 2 months–3 months | 0.85 | ( | 0.43 | , | 1.65 | ) | 0.62 |
|  | 3 months–4 months | 0.73 | ( | 0.29 | , | 1.83 | ) | 0.50 |
|  | 4 months–5 months | 1.79 | ( | 0.77 | , | 4.17 | ) | 0.18 |
|  | 5 months–6 months | - | ( | - | , | - | ) | - |
|  | 6 months–7 months | - | ( | - | , | - | ) | - |
|  | 7 months–8 months | - | ( | - | , | - | ) | - |
|  | 8 months–9 months | - | ( | - | , | - | ) | - |
|  | 9 months–10 months | - | ( | - | , | - | ) | - |
|  | 10 months–11 months | - | ( | - | , | - | ) | - |
|  | 11 months–12 months | - | ( | - | , | - | ) | - |
|  | ≥ 12 months | - | ( | - | , | - | ) | - |
| Five-dose COVID-19 vaccine group | 14 days–1 month | - | ( | - | , | - | ) | - |
|  | 1 month–2 months | 1.48 | ( | 0.17 | , | 13.11 | ) | 0.72 |
|  | 2 months–3 months | - | ( | - | , | - | ) | - |
|  | 3 months–4 months | - | ( | - | , | - | ) | - |
|  | 4 months–5 months | - | ( | - | , | - | ) | - |
|  | 5 months–6 months | - | ( | - | , | - | ) | - |
|  | 6 months–7 months | - | ( | - | , | - | ) | - |
|  | 7 months–8 months | - | ( | - | , | - | ) | - |
|  | 8 months–9 months | - | ( | - | , | - | ) | - |
|  | 9 months–10 months | - | ( | - | , | - | ) | - |
|  | 10 months–11 months | - | ( | - | , | - | ) | - |
|  | 11 months–12 months | - | ( | - | , | - | ) | - |
|  | ≥ 12 months | - | ( | - | , | - | ) | - |
